# Antifungal susceptibility and growth inhibitory response of oral *Candida* species to *Brucea javanica* Linn. extract

**DOI:** 10.1186/1472-6882-13-342

**Published:** 2013-12-04

**Authors:** Mohd-Al-Faisal Nordin, Wan Himratul Aznita Wan Harun, Fathilah Abdul Razak

**Affiliations:** 1 Department of Oral Biology and Biomedical Sciences, Faculty of Dentistry, University of Malaya, Kuala Lumpur 50603, Malaysia

**Keywords:** Antifungal activity, *Brucea javanica*, *Candida* species, Growth inhibitory effect

## Abstract

**Background:**

*Candida* species have been associated with the emergence of resistant strains towards selected antifungal agents. Plant products have been used traditionally as alternative medicine to ease candidal infections. The present study was undertaken to investigate the antifungal susceptibility patterns and growth inhibiting effect of *Brucea javanica* seeds extract against *Candida* species.

**Methods:**

A total of seven *Candida* strains that includes *Candida albicans* ATCC14053, *Candida dubliniensis* ATCCMYA-2975, *Candida glabrata* ATCC90030, *Candida krusei* ATCC14243, *Candida lusitaniae* ATCC64125, *Candida parapsilosis* ATCC22019 and *Candida tropicalis* ATCC13803 were used in this study. The antifungal activity, minimum inhibitory concentration and minimum fungicidal concentration of *B. javanica* extract were evaluated. Each strain was cultured in Yeast Peptone Dextrose broth under four different growth environments; (i) in the absence and presence of *B. javanica* extract at respective concentrations of (ii) 1 mg/ml (iii) 3 mg/ml and (iv) 6 mg/ml. The growth inhibitory responses of the candidal cells were determined based on changes in the specific-growth rates (μ) and doubling time (g). The values in the presence of extract were computed as percentage in the optical density relative to that of the total cells suspension in the absence of extract.

**Results:**

*B. javanica* seeds extract exhibited antifungal properties. *C. tropicalis* showed the highest growth rate; 0.319 ± 0.002 h^-1^, while others were in the range of 0.141 ± 0.001 to 0.265 ± 0.005 h^-1^. In the presence of extract, the lag and log phases were extended and deviated the μ- and g-values. *B. javanica* extract had significantly reduced the μ-values of *C. dubliniensis*, *C. krusei* and *C. parapsilosis* at more than 80% (*ρ* < 0.05), while others were reduced within the range of 2.28% to 57.05%. The g-values of most candidal strains were extended and significantly reduced (*ρ* < 0.05) in relative to the untreated. The candidal population was reduced from an average of 10 x 10^6^ to 6 x 10^6^ CFU/ml.

**Conclusions:**

*B. javanica* extract exhibited *in vitro* antifungal activity against seven oral *Candida* species. The fungistatic and growth inhibiting effects of *B. javanica* extract have shown that it has potential to be considered as a promising candidate for the development of antifungal agent in oral health products.

## Background

In recent years, there has been an increase of reported cases of oral candidiasis. Less pathogenic strains of candidal species were found to be associated with oral candidiasis [1-3]. Although several species *Candida* comprise part of the oral commensal microflora, their emergence may at time cause opportunistic infections among debilitated and immunocompromised hosts [4,5]. The interactions between candida and the host are extremely complex. The conversion from commensalism to parasitism and exuberant growth is usually associated with intraoral environmental changes and/or a diverse array of local, systemic and iatrogenic factors. Growth kinetic is a key virulence factor of most microorganisms including the *Candida* species. *Candida* species have the ability to grow under diverse environmental conditions. Denture and surfaces of the oral mucosa, for instance, may provide an adequate site for *Candida* species to proliferate and establish an infective process. In the oral ecosystem, substantial change in any key environmental parameter that affects bacterial growth can disrupt the natural balance of the microbial component that may lead to opportunistic infection of the host. The overgrowth of opportunistic organism such as candida over other microorganisms can ultimately induce inflammation of the mucosal tissues. This is due to the action of extracellular enzymes of the growing yeast. Different species of candida are variably susceptible to drugs even within the same species, and certain species can develop resistance to common prescribed antifungal agents [6]. Therefore, an effective antifungal treatment initiated at an early stage of infection may contribute to better understanding of the sustainability of these candidal species. Recent appearance of *Candida* species with reduced susceptibility to antibiotics, and spreading issues concerning the safety of chemical preservatives and drugs have prompted researchers to study antimicrobial agents from natural resources. More than 35,000 plants species are being used in various human cultures for medical purposes [7]. There is great demand for agents from natural resources as they are presumed not associated with many side effects [8-10]. *Brucea javanica* L. (Simaroubaceae), a synonym of *Brucea amarissima* is a plant indigenous to China, India, Indonesia, Malaysia, Thailand and Vietnam [11]. The seed and seed oil of this plant have been used in the treatment of warts and corns. In other countries, the bark or root bark of *B. javanica* is a folk remedy for dysentery and verrucous tumor or cancer [12]. It has been reported that *B. javanica* extract exhibited antiproliferative and apoptosis-inducing activity on human carcinoma cells [13]. However, there is a lack of scientific reports that indicate the antifungal activities of *B. javanica* against oral *Candida* species. Hence, this study aimed to investigate the *in vitro* antifungal activity of *B. javanica* aqueous extract against the seven candidal species and the growth inhibitory response of each *Candida* species was evaluated to elicit the efficacy of the extract.

## Methods

### Plant collection and extract preparation

The seeds of *Brucea javanica* L. were introduced by a colleague Mr. Zabidi Mohd Majid, obtained from a rural area in Sekinchan, Selangor. The specimen was scientifically identified by a botanist and the voucher specimen was deposited at the Herbarium of Rimba Ilmu, University of Malaya. Crude extract of the seeds was prepared according to Himratul-Aznita *et al.*[14]. The seeds (100 g) were cleaned in running water and oven-dried at 60-65°C for two days. The dried seeds were homogenized into small pieces prior to extraction using distilled water at a ratio of sample to water of 1:10. The homogenate was boiled at high temperature to one-third of the original volume. The decoction was filtered through a filter paper (Whatman No.1) to remove debris before it was further boiled to a final volume of 100 ml. The decoction was concentrated by freeze drying (EYELA FDU-1200, Tokyo) overnight. The powder obtained was sealed in a sterile Falcon tube and stored at 4°C. Stock solution of the extract was prepared in sterile distilled water at concentration of 200 mg/ml. Following centrifugation (Jouan A14, France) for 10 min at 10,000 rpm, the stock was then diluted to concentrations required for the experiment. The extract was sterilised by filtration using 0.2 μm nylon syringe filter (Milipore, USA).

### Preparation of candidal suspension

Seven oral *Candida* species (*Candida albicans* ATCC 14053, *Candida dubliniensis* ATCC MYA-2975, *Candida glabrata* ATCC 90030, *Candida krusei* ATCC 14243, *Candida lusitaniae* ATCC 64125, *Candida parapsilosis* ATCC 22019 and *Candida tropicalis* ATCC 13803) used in this study were purchased from the American Type Culture Collection (ATCC), USA. These may be a typical representative of the species to which it may be assigned. The candidal stock which was kept frozen in glycerol at −70°C was thawed at room temperature and then aseptically dispersed in 5 ml of Yeast Peptone Dextrose (YPD) broth (BD Difco™) before incubating overnight at 37°C. The suspensions were then centrifuged at 10,000 rpm (10°C) for 5 min to harvest the cells. The supernatant was discarded while the pellet was washed twice with sterile saline (NaCl, 8.5 g/L) and then re-suspended in 5 ml of YPD broth. The turbidity of the suspension was adjusted and standardized spectrophotometrically to an optical density (OD550nm) of 0.144 which is equivalent to 1 × 10^6^ cells/ml or to #0.5 McFarland standard [14].

### Antifungal susceptibility

The antifungal activity of the extract was carried out based on the disc diffusion concept of the Kirby-Bauer sensitivity test [15]. Sterile blank discs of 6 mm diameter were impregnated with a concentration of 100 mg/ml. The discs were air-dried prior to firm placement on the agar surface which had earlier been seeded with the respective candidal. Throughout this experiment, a blank disc impregnated with sterile distilled water represented as negative control while a disc impregnated with a mouth rinse containing 0.12% w/v chlorhexidine digluconate (CHX) represented as the positive control. The volume of the test extracts, positive and negative controls impregnated onto the discs were standardized at 100 μl. All plates were incubated overnight at 37°C (except for *C. parapsilosis* which required incubation temperature of 35°C). The susceptibility of candidal species was determined by the diameter of the growth inhibited zone surrounding the discs. The experiment was carried out three times in triplicate to ensure reproducibility of observations.

### Determination of minimum inhibitory concentration (MIC)

Two-fold microdilution broth method was used to determine the MIC value [16]. The MIC is the lowest concentration of the samples that visually shows absence of growth. 100 μl of YPD broth was dispensed into wells marked as Well 1 (W1) to Well 7 (W7). Following this, 100 μl of stock solution (200 mg/ml) was added into W1 and two-fold serial dilution was repeated for W2 through W5. Hence, the final concentrations of *B. javanica* extract in W1, W2, W3, W4 and W5 were 100, 50, 25, 12.5, 6.25 and 3.13 mg/ml, respectively. CHX was used in place of the plant extract as positive control in W6, while W7 which only contain the mixture of YPD broth and the extract represented the negative control. 20 μl of candidal suspension (10^6^ CFU/ml) was added to W1 through W6, except for W7. Triplicate samples were performed for each test concentration. The microdilution plates were incubated overnight at 37°C (except *C. parapsilosis*, 35°C). Following this, the growth inhibition of the candidal cells in microdilution wells was observed.

### Determination of minimum fungicidal concentration (MFC)

A standard procedure described by Espinel-Ingroff *et al.*[17] was applied to determine the MFC. The MFC criteria value considered in this work was the concentration where no growth or fewer than three colonies were obtained to give an approximately 99 to 99.5% killing activity. Briefly, 50 μl was taken from the wells of the MIC assay in which no indication of growth was observed for all respective *Candida* species, was sub-cultured onto fresh YPD agar plates. The plates were incubated at 37°C (*C. parapsilosis* at 35°C) for 24 to 48 h following which any visible sign of growth.

### Determination of the percentage inhibition of diameter growth (PIDG)

PIDG provides an indication with regards to the strength of antifungal activity of the extract in comparison to the positive control (0.12% w/v CHX). The percentage inhibition of diameter growth (PIDG) values was estimated according to the equation as below [14]:

$$\mathrm{PIDG}\;\left(\%\right)=\frac{\text{Diameter}\;\mathrm{of}\;\text{sample}‒\text{Diameter}\;\mathrm{of}\;\text{positive}\;\text{control}}{\text{Diameter}\;\mathrm{of}\;\text{positive}\;\text{control}}\times \;100$$

### Growth profiles of Candida species

Five millilitre of candidal suspension (10^6^ cells/ml) was dispensed into three sterile conical flasks, each containing 40 ml of YPD broth. 5 ml of sterile distilled water was added to give a total volume of 50 ml in each flask. The flasks were incubated at 37°C (*C. parapsilosis* at 35°C) for 18 h in a shaking water bath to continuously agitate the suspension. The growth of each species was elucidated by viable cell counts (CFU enumeration) which were estimated at 2, 6, 10, 14 and 16 h interval. The cell suspension was first diluted by serial dilution in a nontoxic diluent (e.g. phosphate-buffered saline, pH 7.2-7.4) before plating. Spectrophotometric assay [18] which was based on continuous monitoring of changes in the optical density of cell growth was employed. Cell growth was measured periodically at every one hour interval over a period of 18 h at an on optical absorbance of 550 nm. The growth of different candidal species can be distinguished by measuring the changes of specific-growth rate (μ) and doubling time (g) following equations previously described [19,20]:where, N_t_ represented the number of cells at log phase, N_o_ represented the number of cells at zero time, t_2_ was the time taken to reach plateau, and t_1_ zero time when the cells enter the log phase. Throughout of the study, CHX was used in place of the extract as a positive control.

(i) Specific-growth rate: $\mu =\frac{\mathrm{In}\;\left(N_{t}/N_{o}\right)}{t_{2}-t_{1}}$

(ii) Doubling time: g = log_10_(N_t_/N_o_)/log_10_2

### Growth inhibitory activity of Brucea javanica extract

*Brucea javanica* extract was prepared into stocks of 10, 30 and 60 mg/ml. Five mililiter of each stock concentration was dispensed into sterile conical flasks containing 40 ml of YPD broth, followed by 5 ml of the respective candidal suspension (10^6^ cells/ml) to give a final concentration of 1, 3 and 6 mg/ml of the extract. In a similar manner, the culture flasks were placed in a shaking water bath at 37°C (*C. parapsilosis* at 35°C) and the growth of cells in presence of the extract was measured periodically at every one hour interval over a period of 18 h. Changes in specific-growth rate (μ) and doubling time (g) were calculated and the findings were compared with that of the standard. The inhibitory effect of the extract was also determined based on viable cell counts.

### Statistical analysis

All results were computed and expressed as mean ± standard deviation (SD) from three determinations performed in triplicate (n = 9). Statistical analysis was performed using SPSS software (version 18.0) with analysis of variance (One-Way ANOVA) and post-hoc test Dunnett’s T3 were used to compare the significant difference between the groups. A *ρ*-value of < 0.05 was considered as statistically significant.

## Results

### Antifungal activity, MIC and MFC of extract

The diameter of inhibition zone (DIZ), MIC, and MFC values are presented in Table 1. The DIZ values showed that *B. javanica* aqueous extract had a wide range of antifungal activities over the candidal species tested. Of the seven species, *C. dubliniensis* was the most sensitive (DIZ 22.7 ± 1.0 mm) to the extract, whereas the others were relatively lower in the range of 11.3 to 15.6 mm. *C. dubliniensis*, *C. glabrata*, *C. lusitaniae* and *C. parapsilosis* were more susceptible to the extract (≥ 15 mm) whereas *C. albicans*, *C. krusei* and *C. tropicalis* were resistant (< 15 mm). Thus, 15 mm that represented the effect of the extract on three out of seven *Candida* species was used as a breakpoint in this analysis. The other four species showed extreme deviation from this value. The MIC and MFC values were ranged from 3.13 to 100 mg/ml. Chlorhexidine (CHX)-containing (1.2 mg/ml of 0.12% w/v) mouthrinse which used as a reference exhibited clear zones of inhibition to range between 9.3 to 25.0 mm (MIC and MFC of 1.87^-3^% w/v) (Table 1). It is always possible that the susceptibility of candidal species may not be uniform based on its minimum strength to inhibit the candidal cells.

**Table 1:** **Antifungal activity (DIZ), MIC and MFC of *Brucea javanica *** **L. seeds extract**^**a**^

	**Antifungal properties**^**b**^			
***Candida *** **species**	**DIZ (mm)**	**CHX (mm)**	**MIC values**	**MFC values**
	**(Mean ± SD)**	**(Mean ± SD)**	**(mg/ml)**	**(mg/ml)**
*C. albicans*	13.8 ± 0.8	13.0 ± 0.2	50	100
ATCC 14053				
*C. dubliniensis*	22.7 ± 1.0	11.1 ± 0.3	3.13	12.5
ATCC MYA-2975				
*C. glabrata*	15.6 ± 1.6	9.0 ± 0.5	25	50
ATCC 90030				
*C. krusei*	12.0 ± 1.3	20.2 ± 0.7	50	100
ATCC 14243				
*C. lusitaniae*	15.1 ± 0.8	12.1 ± 0.4	25	25
ATCC 64125				
*C. parapsilosis*	15.0 ± 0.9	25.0 ± 0.2	25	25
ATCC 22019				
*C. tropicalis*	11.3 ± 1.0	19.3 ± 0.3	50	100
ATCC 13803				

^a^Antifungal activity was evaluated by measuring the diameter of inhibition zone (DIZ) of the seven candidal species. The concentration of *B. javanica* seeds extract was standardised at 100 mg/ml. CHX represents a positive control.

^b^Values are determined based on three determinations performed in triplicate (n = 9).

### Determination of PIDG

**Figure 1:**
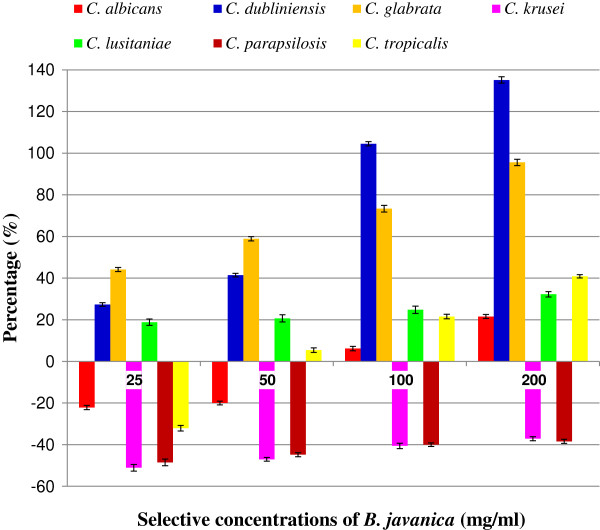
**The PIDGs evaluation which represents the percentage of inhibition of seven candidal species upon exposure with *B. javanica *aqueous extract at respective concentrations of 25, 50, 100 and 200 mg/ml.** The values plotted were expressed as mean ± SD of three determinations performed in triplicate (n = 9).

Based on the PIDG values obtained for all the seven species, *C. glabrata*, *C. dubliniensis*, *C. lusitaniae* and *C. tropicalis* were determined susceptible to *B. javanica* extract, indicating a high PIDG over the control. The rest of the candidal species however, showed comparatively low susceptibility to the extract compared to the control (Figure 1).

### Growth profile of Candida species

**Figure 2:**
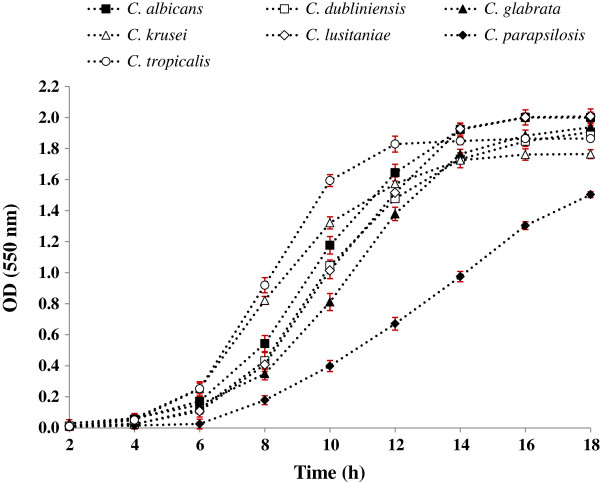
**Normal growth curves of *Candida *species cultured in YPD broth media.** The cell growth signified by the sigmoidal growth curve pattern indicating an orderly increase in cell mass and size. The values are expressed mean ± SD from three independent experiments performed in triplicate (n = 9).

**Figure 3:**
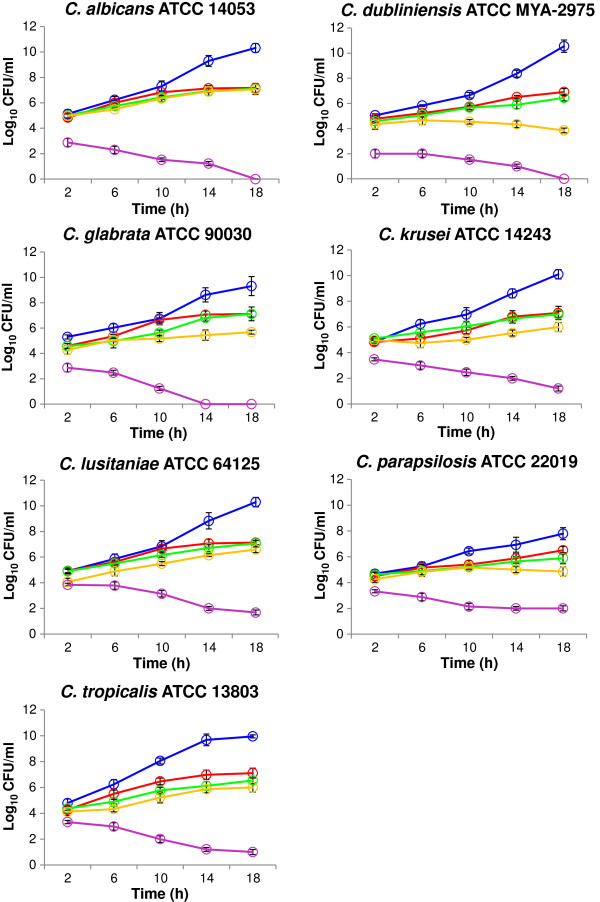
**The population of candidal species under treatment of *B. javanica *extract at 1 mg/ml (red circle), 3 mg/ml (green circle) and 6 mg/ml (yellow circle).** Deviation of cell numbers from the normal (blue circle) indicates the effect of the extract on the growth. CHX was used as a reference (pink circle). Data are mean ± SD of three independent experiments performed in triplicate (n = 9).

Figure 2 displayed the normal growth curves of the seven candidal species cultured under the normal, untreated growth condition. The curves were all sigmoidal, with clear exhibition of the lag, log and stationary phases. Vary durations of the lag and log phases were observed among the different species. In general, about 5 to 7 h was required by the cells to adapt to the normal growth environment before they were ready to proliferate and enter the log phase. *C. tropicalis* showed the highest growth rates (0.319 ± 0.002 h^-1^) indicating high proliferation. The others were in the range of 0.141 ± 0.001 to 0.265 ± 0.005 h^-1^. The doubling time of *C. dubliniensis* (3.330 ± 0.164 h) was observed to be slightly longer than the others which were ranged between 1.816 ± 0.052 h and 2.229 ± 0.037 h. Growth kinetics of the candidal species was also elucidated based on the enumeration of the colony forming units (CFU) (Figure 3). It is clearly shown that the population of candidal species was gradually increased from 4.5 × 10^6^ to 10.0 × 10^6^ CFU/ml over 18 h of incubation.

### Growth inhibitory response of Candida species to Brucea javanica extract

The pattern of growth curves of the candidal species were altered and showed deviations from the normal curves following treatment with *B. javanica* extract. Reduction in cell population was also observed when the candidal cells were cultured at selected concentrations (Figure 3). Although each growth phase is affected by the extract in different ways, the most obvious change induced by exposure of the candidal cells to with the increase in extract concentration, the growth curves were observed to shift to the right due to extension of the lag phases. This effect together with the suppression of cell growth was indicated by the elevated g-values and reduction in μ-values, respectively (Table 2).

**Table 2:** **Changes in the doubling time (g) and specific-growth rates (μ) of the seven candidal species that cultured in the absence (untreated) and presence of *Brucea javanica *** **extract**

***Candida *** **spp.**	**Doubling time (g) & specific-**	**Untreated**	***Brucea javanica *** **L.**		
	**growth rates (μ)**		**1 mg/ml**	**3 mg/ml**	**6 mg/ml**
*Candida*	g (h)	2.403 ± 0.095	2.461 ± 0.083	2.727 ± 0.101	3.021 ± 0.094
*albicans*	μ (h^-1^)	0.263 ± 0.011	0.257 ± 0.008	0.130 ± 0.022	0.112 ± 0.006
	Increase in g	-	1.02-fold	1.13-fold	1.26-fold
	Reduction in μ	-	2.28%	50.57%	57.41%
*Candida*	g (h)	3.330 ± 0.164	2.526 ± 0.124	2.096 ± 0.204	0.271 ± 0.016
*dubliniensis*	μ (h^-1^)	0.251 ± 0.010	0.034 ± 0.012	0.014 ± 0.003	0.002 ± 0.002
	Increase in g	-	0.76-fold	0.63-fold	0.08-fold
	Reduction in μ	-	86.45%	94.42%	99.20%
*Candida*	g (h)	2.229 ± 0.037	3.061 ± 0.049	2.543 ± 0.041	2.843 ± 0.177
*glabrata*	μ (h^-1^)	0.263 ± 0.004	0.249 ± 0.005	0.218 ± 0.004	0.025 ± 0.006
	Increase in g	-	1.37-fold	1.14-fold	1.28-fold
	Reduction in μ	-	5.32%	17.11%	90.49%
*Candida*	g (h)	2.542 ± 0.045	3.109 ± 0.241	1.950 ± 0.096	1.024 ± 0.042
*krusei*	μ (h^-1^)	0.251 ± 0.006	0.030 ± 0.003	0.028 ± 0.002	0.015 ± 0.002
	Increase in g	-	1.22-fold	0.77-fold	0.40-fold
	Reduction in μ	-	88.05%	88.84%	94.02%
*Candida*	g (h)	2.694 ± 0.058	3.425 ± 0.111	2.708 ± 0.046	0.867 ± 0.043
*lusitaniae*	μ (h^-1^)	0.265 ± 0.005	0.238 ± 0.003	0.087 ± 0.005	0.008 ± 0.002
	Increase in g	-	1.27-fold	1.01-fold	0.32-fold
	Reduction in μ	-	10.19%	67.17%	96.98%
*Candida*	g (h)	1.816 ± 0.052	1.615 ± 0.108	1.554 ± 0.098	0.614 ± 0.105
*parapsilosis*	μ (h^-1^)	0.141 ± 0.001	0.017 ± 0.004	0.010 ± 0.002	0.003 ± 0.001
	Increase in g	-	0.89-fold	0.86-fold	0.34-fold
	Reduction in μ	-	87.94%	92.91%	97.87%
*Candida*	g (h)	2.809 ± 0.017	3.967 ± 0.089	3.466 ± 0.108	0.568 ± 0.055
*tropicalis*	μ (h^-1^)	0.319 ± 0.002	0.137 ± 0.003	0.026 ± 0.005	0.005 ± 0.003
	Increase in g	-	1.41-fold	1.23-fold	0.20-fold
	Reduction in μ	-	57.05%	91.85%	98.43%

Values were obtained from spectrophotometric assay and expressed as mean ± SD of three independent experiments performed in triplicate (n = 9).

The growth suppression effect of the extract was found to be concentration dependent. At 1 mg/ml, the μ-values of *C. parapsilosis*, *C. krusei*, *C. dubliniensis* and *C. tropicalis* were significantly reduced by 87.94%, 88.05%, 86.45% and 57.05%, respectively (*ρ* < 0.05). At 3 mg/ml, the μ-values of *C. glabrata* and *C. albicans* were reduced by 17.87% and 50.57%, respectively. At higher concentration of 6 mg/ml, the μ-values of all candidal species were further reduced by more than 90% except for *C. albicans* (57.41%) (*ρ* < 0.05). Meanwhile at 1 mg/ml, the extract causes an increase to the g-values within the range of 0.76-fold to 1.41-fold. At 6 mg/ml however, the g-values significantly fell within the range of 0.08-fold to 1.28-fold (*ρ* < 0.05) compared to the untreated candida. Deviations in the μ- and g-values had led to extension of the lag and log phases. Based on CFU enumeration, the cell population (CFU/ml) of all species was also reduced from an average of 10 × 10^6^ to 6 × 10^6^ CFU/ml.

## Discussion

Despite fluctuations in the external surroundings that may affect the unicellular *Candida* species, these cells respond differently to adverse environmental conditions in order to sustain growth. At present, there are serious efforts to discover compounds with promising antimicrobial activities from plants [21,22]. *B. javanica* extract has been used externally to treat vaginal trichomoniasis and various fungal infections [23], also exhibited antiproliferative activity on human carcinoma cells [13]. In this study, the extent of antifungal activity of this extract was further studied on seven species of oral candida. The efficacy of the extract on the growth profiles of the organisms was determined based on continuous monitoring of changes in the optical density of fungal growth over time. Similar approach was employed to analyze the growth characteristics of various filamentous fungi and oral bacteria [18,24]. High reproducibility and accuracy of the method have been reported. Sub-minimal inhibitory concentrations (sub-MICs) were used throughout the experiment to ensure that the observed reduced optical absorbance was not an attribution of cell death.

From the Kirby-Bauer sensitivity test, *B. javanica* extract was found to exhibit varying degree of antifungal activity towards different species of candida. The percentage inhibition of radial growth (PIRG) [25] was applied with some modification. In this *in vitro* study, the results were incorporated into the formula for PIDG determination. Based on the percentage plotted in Figure 1, *C. glabrata*, *C. dubliniensis* and followed by *C. lusitaniae* were highly affected by the extract of *B. javanica* which outstrips the CHX (positive control). This was indicated by the positive values of PIDG. The PIDG for *C. albicans* however outstrips the positive control at high concentration of 200 mg/ml, suggesting that the effectiveness of the extract on each candidal cell was dose-dependent. The other candidal species were less affected by the control and this explains the effectiveness of CHX as a reference in many clinical trials.

In normal growth condition, the duration of the lag phase (the period until the first significant change in OD) of candidal species was ranged from 5 to 7 h indicating that different species in the genus *Candida* have different reproducibility. When curves of growth in the presence of increasing concentrations of *B. javanica* extract were studied, both parameters; the doubling time (g) and specific-growth rates (μ) of seven candidal species were affected compared with those of the extract-free (untreated) growth media. The extract exhibited significant reduction towards the growth of all seven candidal tested (*ρ* < 0.05). The g-values of most candidal species were also extended and reduced by 90%, specifically at concentration of 6 mg/ml. The extended lag phases strongly suggest that the extract has successfully suppressed the cells and exerted fungistatic effect on the candidal species. This indicates that candidal cells grown in the presence of the extract could experience environmental stress, which could influence its ability to use nutrients efficiently and disrupt their normal biological functions [19]. The need of putative enzymes may also be suppressed. The cells were not able to grow as normal since they were not able to attain the predetermined size and volume required to enable them to divide successfully. The cells may deactivate its metabolism while waiting for the environment to revert back to normal.

It was shown that the higher extract concentrations, the longer the lag phase of the growth curves. This effect was previously observed for candidal species when growth curves based on capacitance were obtained [26]. The presence of extract decreased the germination and elongation rates of spores and germinated spores, respectively. Therefore, the critical turbidity was delayed, resulting in longer lag phases. Upon the addition of *B. javanica* extract into the growth environment, the log phases of candidal species were also reduced. Besides, the internal system of some candidal cells might have been disrupted by the presence of *B. javanica* extract and this clearly explained by the reduction of cell population in Figure 3. Considering the candidal species as a part of the normal flora in human oral cavity, it is acceptable to allow the yeast cells to grow at minimal rate, or at least to diminish the overgrowth of candidal species over the other microflora.

## Conclusion

In conclusion, this study showed that the crude *B. javanica* extracted from the seeds possessed antifungal activity against the seven candidal species tested. The efficacious of the extract was elucidated by the growth inhibitory response and fungistatic activity on candidal species. The extract exerted fungicidal activity at a high concentration, indicating the effect is concentration dependent. This implies that it has potential to be developed as an alternative of antifungal agent which later can be applicable in oral health products. Further study is necessary to access the effects of the extract on oral mucosal cells. Isolation and characterization of chemical constituents of the extract would also be necessary to identify the active component with potential for clinical use.

## Competing interests

The authors declare that they have no competing interests.

## Authors’ contributions

All authors MFN, WHA and FAR took part in experimental design, acquisition of data, statistical analysis, interpretation and writing of the manuscript. All authors read and approved the final version of manuscript.

## Pre-publication history

The pre-publication history for this paper can be accessed here:

http://www.biomedcentral.com/1472-6882/13/342/prepub
